# Risk factors associated with home care safety for older people with dementia: family caregivers’ perspectives

**DOI:** 10.1186/s12877-023-03893-3

**Published:** 2023-04-07

**Authors:** Guo Yin, Siting Lin, Linghui Chen

**Affiliations:** 1 School of Nursing, Sanquan College of Xinxiang Medical University, Xinxiang City, Henan Province China; 2 School of Nursing and Health, Nanfang College, Guangzhou, Guangzhou City, Guangdong, China; 3 Cicely Saunders Institute of Palliative Care, Policy & Rehabilitation, Florence Nightingale Faculty of Nursing, Midwifery & Palliative Care, King’s College London, London, UK

**Keywords:** Dementia, Family caregivers, Safety, Home care

## Abstract

**Background:**

Many older people with dementia currently prefer home care; however, homes are neither professionally designed nor regulated like health care facilities, and home care is more prone to safety incidents. Many studies have examined home care safety for older people with dementia. However, factors contributing to safety incidents in home care have not been adequately considered. This study explored the risk factors for home care safety for older people with dementia based on the perspective of family caregivers.

**Methods:**

This study used a qualitative research approach; a total of 24 family caregivers were interviewed face-to-face and semi-structured from February 2022 to May 2022, and the Colaizzi seven-step phenomenological research method was used to analyze the data and refine the themes.

**Results:**

Safety risks in home care for older people with dementia stem from five areas: poor health of older people with dementia, dementia symptoms, unsafe home environment, the insufficient caring ability of family caregivers, and lack of safety awareness of family caregivers.

**Conclusion:**

The risk factors for home care safety for older people with dementia are complex. And as the primary caregivers of older people with dementia, the caregiving ability and safety awareness of family caregivers primarily determine the safety of home care for older people with dementia. Therefore, when addressing home care safety for older people with dementia, the focus should be on providing targeted education programs and support services for family caregivers of older people with dementia.

## Background

Dementia is a degenerative disease of the central nervous system with progressive cognitive and memory impairment as its main clinical manifestations [1]. China has the highest number of older people with dementia (OPwD) in the world, with 20% of the world’s dementia population [2]. With the aging of the Chinese population, the number of older people with dementia continues to increase. The number of OPwD in China will increase from about 9.5 million in 2015 to 16 million by 2030 [3]. However, the availability of specialist medical care services is minimal compared to the significant increase in the total number of OPwD [4]. Many long-term care system facilities do not accommodate OPwD due to a lack of skilled nursing staff and limited space [5]. In addition, older people also have a strong cultural preference for aging in place [6]. Social customs, cultural expectations, and legal obligations also emphasize that caring for older adults is the family’s responsibility [7]. Therefore, most OPwD choose home care [8]. Home care is the process of enabling OPwD to maintain daily living in their home through the help of caregivers [9].

The Chinese government has established community-based home care services, such as daycare centers, respite care services, and in-home dementia care services provided by professional caregivers; however, these services are still in the exploratory stage [10]. Furthermore, only a small proportion of OPwD from high-income families have access to formal home care services provided by skilled caregivers [8, 11]. As a result, in China, 85% of OPwD are primarily cared for by family members at home [12], and adult children or spouses of OPwD are the primary caregivers in the family [11]. These family caregivers primarily provide daily care such as dressing and eating, bathing, and providing social and emotional support for OPwD [2].

Home care can be helpful or threatening for OPwD who are cognitively and mobility impaired. Because of the fact that the home environment is designed for life, not health care delivery [13]. Homes are neither professionally designed nor regulated like medical institutions [14]. Therefore, home care’s likelihood of adverse events may be higher than professional healthcare [15]. Identified Home safety issues for OPwD include falls, wandering, burns, drowning, food safety, choking, and medication errors [16–19].

Numerous studies have examined safety in home care, but few studies concerning the causes of unintentional home injury in OPwD, and the factors that lead to home care safety incidents have not been fully considered. Family members are often the direct caregivers of OPwD and can help identify safety risks [20]. Therefore, the purpose of this study is to explore the risk factors for home care safety for OPwD through in-depth interviews with family caregivers. This will serve as a basis for the further study of the formulation of preventive measures, thereby reducing the incidence of safety incidents of home care for OPwD.

## Methods

### Study design

This study uses a phenomenological research approach to explore risk factors of home care safety for OPwD [21]. This approach allows for the study of the details and essential meaning of participants’ lived experiences through the interpretation of texts and participants’ interpretations of their experiences [22].

### Participants

Participants were recruited from three communities in Guangzhou between February 2022 and May 2022. To ensure access to more thorough and reliable information, family caregivers of OPwD from different socio-demographic backgrounds (different ages, genders, relationships with OPwD, education level, and OPwD included different dementia severity, age, and comorbidity) were selected using purposive sampling. Family caregivers recruited met the following inclusion criteria: (1) Care recipients have a dementia diagnosis record and are cared for at home; (2) The family member of OPwD; (3) Continuously undertake home care tasks for ≥ 6 months. (In order to have enough care experience to share).

Recruitment was stopped when the data was saturated, which means no new information and themes emerged from more interviews [24]. In this study, no new themes emerged when interviewing the 22nd family caregiver. Two additional family caregivers were interviewed to determine that the data reached saturation. Finally, 24 family caregivers of OPwD were recruited from February to May 2022, named by the numbers P1-P24. General information on family caregivers is shown in Table 1.

**Table 1:** Demographic characteristics of Family caregivers and Care recipient in the study (n = 24)

Family Caregivers								Care recipient			
No.	Age	Gender	Education	Residence	Relationship with older people with dementia	Average daily care (hours)	Number of years of care (year)	Age	Gender	Number of years with dementia	Other diseases
P1	43	Male	Junior college	City	Grandparents and Grandchild	5	2	78	Female	8	Hypertension
P2	48	Female	High school	City	Mother and Daughter	24	0.5	78	Female	2	Hypertension; Hyperlipemia; Coronary Heart Disease
P3	26	Female	University	Countryside	Grandparents and Grandchild	4	2	70	Female	2	Hypertension
P4	55	Male	Junior college	City	Father and Son	1	10	80	Male	10	Dermatitis
P5	50	Female	Junior high school	Countryside	Mother and Daughter	24	5	74	Female	5	Hypertension
P6	28	Female	University	City	Grandparents and Grandchild	3	4	76	Female	4	None
P7	58	Male	Primary school	Countryside	Father and Son	2	6	88	Male	5	Hypertension
P8	50	Male	University	City	Mather and Son	2	20	72	Female	3	Hypertension
P9	45	Female	High school	City	Mother and Daughter	3	1	80	Female	1	Hypertension
P10	41	Female	Junior high school	Countryside	Mother-in-Law	3	1	62	Female	1	None
P11	48	Female	Junior high school	City	Father and Daughter	15	6	73	Male	6	Hypertension
P12	50	Female	Junior high school	Countryside	Father and Daughter	12	2	74	Male	2	None
P13	36	Female	University	City	Mother and Daughter	4	2	73	Female	2	Hypertension
P14	40	Female	High school	City	Mother-in-law	2	3	70	Female	3	Arthritis
P15	52	Female	Primary school	City	Mother and Daughter	8	3	85	Female	5	Coronary Heart Disease;Rheumatoid Disease
P16	36	Male	High school	Countryside	Father and Son	3	4	61	Male	4	Gastritis
P17	38	Female	High school	Countryside	Grandparents and Grandchild	7	3	88	Female	5	Hypertension
P18	27	Female	University	City	Grandparents and Grandchild	4	5	86	Female	10	Gastritis;Hypertension
P19	45	Female	Primary school	City	Grandparents and Grandchild	8	12	90	Female	2	Gastritis; Heart Disease
P20	26	Female	University	Countryside	Mother-in-Law	8	2	65	Female	2	None
P21	49	Female	Junior high school	Countryside	Mother-in-Law	5–6	4	90	Female	3–4	Hypertension
P22	50	Male	Junior high school	Countryside	Mather and Son	3	10	80	Female	10	Hypertension;Diabetes
P23	21	Female	University	Countryside	Grandparents and Grandchild	3–4	4	67	Female	4	Hypertension; Diabetes
P24	46	Female	Junior high school	Countryside	Mother-in-Law	1	6	82	Male	4	Hypertension

### Data collection

This study collected data through face-to-face, semi-structured, in-depth interviews. Based on the consent of the interviewees, the entire interview was recorded. During the interview process, the researcher made flexible adjustments to the order and manner of questioning according to the interview outline and the actual situation of the interviewees, followed up on valuable questions appropriately, and respected the expressions of the interviewees without leading or implying. The location of the interview was chosen to be the interviewee’s home, and the interview lasted 30–40 min.

The initial interview outline was developed based on the literature on home care safety and advice from two geriatric care specialists and one community care specialist. The interview questions were revised based on pre-interviews results with two family caregivers, and the final interview outline is presented below.

1. Can you tell us about your experiences caring for OPwD?
2. What are the causes of home care safety incidents for OPwD?
3. What other factors do you think to threaten the safety of home care for OPwD?
4. What measures have you taken to improve home care safety for OPwD?

### Data analysis

The interview recordings were transcribed and collated into text by the first and second authors (GY and SL) within 24 h after the interviews were completed. The recordings were repeatedly listened to for verification. In this study, the Colaizzi seven-step method of phenomenological research was used to guide the data analysis [23]. Data analysis was conducted respectively by the two researchers (GY and SL), including repeatedly analyzing and reading transcripts, extracting significant statements, coding frequently occurring and meaningful ideas, pooling the coded ideas, identifying similar ideas, and analyzing statements of significance and refining themes. Finally, the three researchers (GY, SL, and LC) compared and discussed the data analysis results and defining final themes. Unclear statements in the recordings were verified with the interviewees through telephone contact. In addition, the interview transcripts and themes also confirmed with the interviewees to ensure that the interview transcripts are a true record of their views.

Although the interviews and data analysis was conducted in Chinese, the quotes presented in the study were first translated into English by the researchers (GY and SL), then checked and back-translated into Chinese by another researcher (LC) for comparison with the content in the original transcripts to ensure that no bias in the data was caused during the translation process.

### Ethics

This study has been approved by the Academic Ethics Committee of Nangfang college of Guangzhou (Permit No. 2,022,052,702). This study was performed in accordance with the ethical standards of the Declaration of Helsinki. The purpose and content of the study and the principle of confidentiality were explained in detail to the interviewees before the interviews. Each interviewee signed informed consent forms. In order to protect the privacy of the participants, the actual names of the interviewees were replaced by numbers.

## Results

The Interview results identified five themes related to the safety risks of home care for OPwD: poor health of OPwD, dementia symptoms, unsafe home environment, the insufficient caring ability and lack of safety awareness of family caregivers. The main themes and subthemes of the interview are shown in Table 2.

**Table 2:** The main themes and subthemes of the interview

Main Themes	Subthemes	Representative Case	Condensed meaning unit	Additional Case
**Poor health of older people with dementia**	Decreased body function	P14	“weakened legs”	P11: “When my father went to the bathroom at night, his legs were weak and he didn’t stand still, and his head accidentally hit the doorknob, causing a brain hemorrhage.“
		P22	“presbyopia”	
		P9	“ate fast and choked”	P19: “Grandma is old, her digestion and chewing ability will be poor, and it is easy to cause choking. One day when she was eating, she was in a hurry and choked, her food got stuck in her throat and she couldn’t swallow it…”
	Comorbid chronic diseases	P10	“severe anemia”	P1: “Grandma has high blood pressure and has had three falls at home due to dizziness caused by high blood pressure.“
**Dementia symptoms**	Memory Loss	P1	“forgot to take antihypertensive drugs”	P6 “When she takes medication, she needs to be supervised, otherwise she will accidentally take or miss the medication.“
		P7	“often forgets to take it”	
		P24	“he couldn’t remember where he was going, what he was going to do, where his home was”	
		P24	“he forgot to turn off the gas stove”	
	Cognitive impairment	P23	“she doesn’t know she is doing things that can hurt herself”	
	Behavioral abnormalities	P13	“she is agitated”	P15: “My mom would exhibit some particularly aggressive behavior, accidentally scratching her arm a few times when agitated, and knocking over some things.“
	Communication barrier	P8	“She was not able to inform us”	P21: “My mother is disabled and can’t speak, so she can’t tell us when something happens.“
**Unsafe home environment**	Safety hazards in the home environment	P14	“the floor is very slippery	P5: “Because it was night, the lighting in the house was insufficient, and his eyesight was poor, so he couldn’t see the road clearly and fell down.“
		P22	“the living environment is not very comfortable, and the lighting is inadequate”	
	Low utilization rate of home security facilities	P15	“the facilities in our home did not meet her needs”	P22: “I try to reduce clutter in the house, make sure the house is tidy to prevent the elderly from falling, and nothing else improves.“
**Insufficient caring ability of caregivers**	Heavy care burden for caregivers	P21	“I was going to cook for her and left her alone in bed”	P14: “Since we are all under retirement age and have to work, we cannot provide 24-hour supervision and care for my mom.“
	Poor supervision of caregivers	P3	“I was away for too long…she lay in bed for too long…she had pressure sores on her back”	P6: “Once my grandmother was home alone and unattended, she ran out of the house and didn’t know the way home, thus she got lost…”
	Poor economic conditions of caregivers	P16	“his financial burden is heavy”	
**Lack of safety awareness of caregivers**	Caregivers lack safety knowledge	P9	“I didn’t know anything about home safety”	P13: “I think it is mainly because we don’t know what factors will threaten the elderly, which makes the elderly have a high risk of danger at home.“
		P16	“we don’t have enough safety awareness”	
	Caregivers inaction	P4	“I have not learned about home safety for older people with dementia in any other ways”	P23: “I didn’t learn about home care for older people with dementia through other means. My family and I take care of the elderly according to our daily life experience.“
		P8	“I don’t actively search for knowledge”	P18: “I didn’t take the initiative to learn something about home care and safety for people with dementia. Because I am usually busy, I don’t have time to learn about it, but I understand the importance of this knowledge now. If there are related knowledge dissemination activities in the community in the future, I will definitely attend them, and I think they are very useful.“
		P14	“I didn’t take the initiative to learn about home safety”	
	Lack of reflection by caregivers	P15	“My mother had a bed fall… and put anti-collision tape on the corner of the table”	
	Lack of social support	P23	“I have not received professional guidance from professional medical staff or community nurses”	P14: “Currently there is no access to knowledge on how to take care of elderly people with dementia. I hope that the community can provide some help and guidance to families with older people with dementia at home.“

### Theme I poor health of OPwD

#### Decreased body function

With the increase of age, the body function of OPwD deteriorates, resulting in reduced muscle strength, balance and coordination, vision loss, and pharyngeal sensory function decline, leading to home safety incidents.

*“My mother fell in the bathroom last year due to her weakened legs; I didn’t know what to do when I saw her fall, I didn’t dare to pick her up straight away, so I had to call 120 to give first aid.“* (P14).

*“My mother’s vision is declining, she has presbyopia, she cannot see clearly. She once missed the edge of the step and fell down the stairs.“* (P22).

*“One day at dinner, my mother ate fast, and then she choked, the food got stuck in her throat, and she couldn’t swallow it. We were very nervous at the time, and then we kept slapping her on the back to recover. “*(P9).

#### Comorbid chronic diseases

Most OPwD have one or more chronic conditions. Chronic illnesses such as anemia can cause older people to lose their balance or faint.

*“It was preparing for dinner when my mother-in-law suddenly fainted in the dining room; luckily, we were by her side and rushed her to the hospital, where the doctor examined her and found that it was caused by severe anemia.”*(P10).

### Theme II dementia symptoms

#### Memory and cognitive impairment

Memory loss becomes the biggest barrier to medication safety for OPwD. OPwD often have unsafe behaviors such as remembering the wrong time to take medication, forgetting to take medication, remembering the wrong type of medication, or taking medication repeatedly.

*“My grandmother once forgot to take antihypertensive drugs, which caused her blood pressure to be uncontrolled. She suddenly fainted at home. We were not around her at that time, and she gradually woke up on her own.“*(P1).

*“My father is old and has high blood pressure. He needs to take medicine regularly, but he often forgets to take it.”* (P7).

Due to their impaired memory and disorientation, OPwD easily get lost when they go out alone.

*" My father-in-law felt sick and went to see a doctor by himself. On the way, he couldn’t remember where he was going, what he was going to do, where his home was, and finally, he contacted us with the help of the police.“*(P24).

With memory loss, OPwD are more forgetful of life events and often cannot remember specific tasks in progress, such as forgetting to turn off the gas or the power to the home. Using water, electricity, and gas threatens home care safety for OPwD.

*“My father-in-law has a bad memory. Once he forgot to turn off the gas stove after cooking.”* (P24).

Cognitive impairment makes OPwD lack the awareness and ability to recognize risky behaviors.

*“Grandma has had experiences of wandering, but she didn’t go very far. Fortunately, she was found. When she’s sober, she would say sorry, I got you into this trouble, but when she is not sober, she doesn’t know she is doing things that can hurt herself.”* (P23).

#### Behavioral abnormalities

Psycho-behavioral symptoms in OPwD may cause them exhibit aggressive behaviors such as yelling, destroying objects, self-harm or abusing others.

*“My mother is sometimes agitated; when she is agitated, she will scratch her arms and knock over objects such as glasses. There were also times when she was frightened by loud noises outside, making her feel scared and agitated.“ (P13)*.

#### Communication barriers

Communication barriers is a common symptom of OPwD [24]. Due to communication barriers, OPwD may not be able to express their symptoms and needs correctly, which can prevent caregivers from promptly identifying OPwD risk conditions.

*“One night, my mother was not feeling very well. We didn’t notice that she was unwell until we noticed that she couldn’t sleep. When I asked her, she couldn’t express clearly. Then we thought it was not normal, so we took her to the hospital and found out that her blood pressure was high. She was demented and was not able to inform us when she was unwell.“* (P8).

*“My grandma had a bed fall, but she couldn’t speak, so she couldn’t inform us in time when something happened.“* (P17).

### Theme III unsafe home environment

#### Safety hazards in the home environment

The home environment of OPwD is a potential safety hazard. Slippery floors caused by water in the bathroom, lack of handrails, and inadequate room lighting can all contribute to OPwD falls.

*“My dad was in the shower; he slipped and fell.“* (P12).

*“The weather in the south is very humid. When it comes to the wet season, the floor is very slippery, and the old people are prone to falls.*“ (P14).

*“Because we live in the countryside, the living environment is not very comfortable*, an*d inadequate room lighting*, *it makes it easy for some accidents to happen. My mother has had falls and missed the edge of the step when went upstairs at home.“* (P22).

The use of electrical appliances is also a major safety hazard faced by OPwD.

*“Once my mother cooked porridge at home, the electric pot might have a little electricity leakage. When she stirred the porridge with the iron spoon, she was electrocuted and burned by the porridge.”* (P22).

#### Low utilization rate of home security facilities

Safety facilities (such as bed rails, anti-slip mats, and bedside alarms) which can reduce the risk of accidental injury in OPwD. However, most family caregivers have low utilization of safety facilities for OPwD.

*“My mother had a bed fall because of our negligence… In addition, the facilities in our home did not meet her needs… I didn’t know the bed rail at that time, so I didn’t install it.”* (P15).

### Theme IV Insufficient caring ability of family caregivers

#### Heavy care burden for family caregivers

Family caregivers were busy with the daily care of older people with dementia. Family caregivers had a heavy caregiving burden, were less concerned about the safety of OPwD, and neglected to prevent adverse events in home care.

*“Mainly because I was going to cook for her and left her alone in bed, and she accidentally fell out of bed and bled from her forehead.“* (P21).

#### Poor supervision of family caregivers

Family caregivers cannot take care of OPwD 24 h a day due to work and other reasons. When family caregivers are away from OPwD, they did not take relevant protection and preparations. Many caregivers reported that OPwD had accidents at home due to OPwD being unattended and their negligence.

*" As I was away for too long and no one was home to look after her, she lay in bed for too long without turning over, and by the time I got home in the evening, she had pressure sores on her back.“* (P3).

*“She ran out of the house while I was sleeping, and it took a day to find her. Mostly because I was careless and forgot to lock the door.“* (P20).

#### Poor economic conditions of family caregivers

The cost of implementing solutions can increase the financial burden on family caregivers, which can discourage financially disadvantaged caregivers from making improvements to their home environment.

*" My brother, my sister, and I each take turns taking care of him for four months a year. When my father lived at my brother’s house, he fell while using the toilet. The bathroom in my brother’s house is a squat toilet…I reminded my brother to rectify it, but he didn’t change it …I think my brother is reluctant to renovate the bathroom because he thinks it is too expensive, and his financial burden is heavy. He has two children who are going to school, and they all need to spend money.“* (P16).

### Theme V lack of safety awareness of family caregivers

#### Family caregivers lack safety knowledge

Some family caregivers of OPwD have insufficient ability to identify safety problems at home and have insufficient knowledge to deal with safety problems.

*“When my mother was hospitalized, I had asked some knowledge about disease care, but I didn’t know anything about home care safety.“* (P9).

It is challenging for family caregivers to recognize risk factors for OPwD at home because of their lack of safety awareness and knowledge.

*“I think we don’t have enough safety awareness, and there are some risk factors in the house that we usually find difficult to notice.”* (P16).

#### Family caregiver’s inaction

Some family caregivers do not take the initiative to gain knowledge about home safety for OPwD.

*“I take care of my father based on my experience. I have not learned about home safety for older people with dementia in any other ways.“* (P4).

*“I mostly learn about home safety for older people with dementia through short videos, but there are so few relevant videos now, and I don’t actively search for knowledge, so I don’t know much about some home safety yet.“* (P8).

*“I didn’t take the initiative to learn about home safety because I was usually busy and didn’t have time to learn about it.“* (P14).

#### Lack of reflection by family caregivers

Some interviewees were still unaware of the actual home safety hazards when OPwD had an accident at home. Many interviewees reported that OPwD had the bed falling incident, but they still did not install bed rails after the incident. They do not reflect on the causes of accidents and injuries that have occurred in OPwD.

*“Since she fell in the bathroom, we wiped the bathroom floor promptly every time.“* (P1).

*“My mother had a bed fall…I have improved the home environment, installed some handrails at home, and put anti-collision tape on the corner of the table.”* (P15).

#### Lack of social support

The prevention of unintentional injuries to OPwD relies heavily on support from the relevant professional medical staff or community workers. However, most family caregivers of OPwD reported that they have little opportunity to receive professional care guidance.

*“I have not received professional guidance from professional medical staff or community nurses. The care of the elderly is mainly based on my experience.“* (P23).

## Discussion

This study examines the safety risk factors of home care for OPwD from the perspective of the family caregiver, and the findings will contribute to a deeper understanding of the safety risks of home care for OPwD and provide insights into strategies to address home care safety risks.

The safety of home care for OPwD is vulnerable to many potential risks, particularly the coexistence of dementia disease and physical aging; as demonstrated by previous studies, **dementia disease causes cognitive, memory, and orientation deficits in older adults, which leads to risky behaviors such as wandering, disorientation, and falls** [25], and when accompanied by some other risk factors such as poor vision, hypotension, muscle weakness, and medication side effects, it increases the likelihood of safety risks for OPwD [26]. Especially when OPwD have one or more chronic conditions, cognitive and memory impairment can lead to medication safety issues such as medication errors, missed doses, and repeated doses [27]. The risks of taking medication may outweigh the benefits because of the challenges of medication safety and adherence [28].

In addition, unlike other diseases (e.g., disability), OPwD not only degenerate in terms of independent living ability, but also experience psycho-behavioral symptoms such as behavioral aggression, neuroticism, and apathy [29]. The appearance of psycho-behavioral symptoms aggravates the cognitive impairment of OPwD, and **pose a threat to their safety and those around them** [30].

Although there is no cure or effective prevention strategy for dementia, early and timely intervention can delay the progression of dementia and reduce cognitive, behavioral, and psychiatric impairment and improve safety risk management in OPwD [31]. However, this study found that no **family caregivers intervened with dementia symptoms in OPwD.** Dementia is considered a normal part of aging due to the misconception of the Chinese public [32]. Interventions and treatments for dementia are considered pointless [33], and the disease is allowed to progress. According to statistics, only 21% of dementia patients in China are treated [34]. This leads to a continuous deterioration of symptoms in OPwD.

On the other hand, OPwD are more reliant on their family caregivers to manage them because of their diminished capacity for self-management. Family caregivers are the primary caregivers for dementia patients, and their knowledge of the disease’s symptoms, progression, and care needs determine disease management and home care safety for OPwD [35]. But as other researchers have found, the majority of family caregivers lack the knowledge and abilities required to care for OPwD [36–38]. Family caregivers are often unprepared for their caring role [39].

In this way, the home care environment constructed by family caregivers for OPwD is concerning. The current home care environment for OPwD is not customized for them, and home care settings lack uniformity [40]. This study found numerous unsafe items in various areas of the home environment for most OPwD, which directly contributes to the frequency of safety incidents in OPwD. Dementia impairs judgment and insight, hence OPwD exhibit weak risk aversion in dangerous home circumstances [41]. Additionally unable to recognize safety hazards and seek help, OPwD may have behavioral impairments (wandering) [42]. Specifically, behavioral impairment has been demonstrated to be a significant risk factor for falls in OPwD. [25]. This also places OPwD at a higher risk of safety in an unstable home environment compared to cognitively healthy older adults [43]. Unstable, frequently changing, and overly cluttered home environments also put OPwD at risk for psycho-behavioral problems, such as aggressive behavior [44]. Therefore, creating a safe care environment for OPwD is considered a prerequisite for home care [45–47].

There are valid and reliable screening tools for home environment risk factors [48, 49], and the use of a home environment safety risk screening form is a time-saving and comprehensive method for assessing the home environment of OPwD. Unfortunately, home environment assessment and modification are not valued by some family caregivers, as evidenced by other studies [50]. Jiang et al. (2017) indicates that family caregivers believe that older adults to “do less and rest more” is a sign of filial piety, so family caregivers are more likely to limit the activities of OPwD to ensure their safety than to reduce or prevent adverse events such as falls by improving the home care environment [50]. This study indicated that family caregivers tend to underestimate the dangers posed by dementia disease risk factors and the safety problems associated with OPWD. When dangers are miscalculated, some preventive measures are not performed [51]. It is noteworthy that family caregivers who do not perceive any home care safety risk for OPwD are not likely to seek supportive solutions proactively.

Given the above, this also reveals that the primary issue in home care safety for OPwD is the lack of awareness of dementia and home care risks among family caregivers, which also stems from inadequate education of family caregivers, in line with the findings of Tudor (2017) [14]. China currently lacks educational programs and support services for OPwD and their family caregivers [12]. On the other hand, dementia is considered a stigmatizing illness due to traditional Chinese culture [52]. Zou et al. (2017) found that family caregivers who view dementia as a “stigmatized mental illness” were reluctant to mention dementia-related concerns and seek assistance [53]. Most importantly, Zhao et al. (2022) revealed that even primary care professionals lack confidence and expertise in dementia management [54]. Due to these circumstances, the majority of family caregivers lack the chance or access to expert dementia care information [37]. Most family caregivers rely on their own life experiences to care for OPwD, but some of these experiences do not match the safety standards of home care for OPwD and may pose safety risks to them.

For this reason, training and education of family caregivers should be given priority. A Seattle protocol in the United States provides a systematic approach to training community caregivers to teach family caregivers to recognize environmental or disruptive events and develop more effective responses [55]. This program has been successful in reducing home safety risks for OPwD. China has currently established a family physician contracting system [56], this systematic approach can be implemented on family physicians, and encourage family physicians to provide educational and training services to family caregivers of OPwD while conducting consultations. When formal caregiving support is limited, some studies suggest peer support for family caregivers of OPwD [57–59]. Peer support is described as assistance from a person in a similar or identical circumstance to the family caregiver [60]. It enhances experience sharing, decreases caregiver uncertainty, and improves caregiver coping with daily living and psychosocial symptoms in OPwD [61]. Consequently, a peer-to-peer social organization can be established in the community to enable caregivers to acquire safety management strategies for home care of OPwD through regular peer-to-peer exchanges.

Another important finding of this study is that family caregivers’ perceptions of safety risks are complex and influenced by multiple factors. Even though caregivers are aware of some safety risks for OPwD, **some factors prevent family caregivers from taking action.** As the results of this study show that caregivers’ motivation to modify harmful home environments is limited by their financial situation. Typical health care delivery structures do not support this, lack of financial resources is a barrier for caregivers to implement home modifications [62]. On the other hand, due to the one-child policy in China, the majority of family caregivers do not have siblings to share the obligation and hardship of caring for their parents with dementia [63]. The multiple roles of caregivers (parents, caregivers, employees) and the difficulty of coordinating work and home caring make home care more challenging [38]. The Supervision of family caregivers has been identified as a crucial behavioral element that can help minimize unintentional injuries in OPwD [64]. However, when family caregivers have limited financial and time resources, both tangible (bed rails and anti-slip mats) and intangible (caregiver supervision) measures to prevent safety risks in OPwD would be absent.

It is also challenging for family caregivers to find effective support and assistance due to the restricted availability of care services for OPwD and the lack of awareness and information among family caregivers about available care services [65–67]. This emphasizes another area of targeted intervention in which specific measures should be implemented to ameliorate the situation, such as expanded availability of healthcare facilities and community-based care services for dementia diseases, with attention to the affordability of services. Furthermore, it is vital to aggressively publicize the availability of support services and to offer effective routes through which family caregivers may access them.

### Limitations and Recommendations

There are some limitations to this study that must be acknowledged. First, all data were collected in Guangzhou, China. This means the study provides insights into home care safety for a limited range of family caregivers of OPwD. On the other hand, this study only identified risk factors for home care through family caregivers of OPwD. If future studies include health professionals, other safety risk factors may emerge. In addition, although data saturation was considered to be achieved. However, due to the nature of qualitative research, this study may not cover all situations. Therefore, a broader mix of quantitative and qualitative studies are needed in the future, which will contribute to a deeper understanding of the risk factors in home care safety for OPwD. Additionally, although analyses were conducted in Chinese rather than English, translations were checked by three researchers fluent in Chinese and English, so the results were not affected by this.

## Conclusion

The risk factors for home care safety for OPwD are complex. Physical health status of OPwD, dementia symptoms, home environment, family caregiver caregiving ability and safety awareness all threaten the safety of home care for OPwD. These findings provide directions for home safety interventions for OPwD. It is also clear that as the primary caregivers of OPwD, the caregiving ability and safety awareness of family caregivers primarily determine the safety of home care for OPwD. Therefore, when addressing home care safety for OPwD, the focus should be on providing targeted education programs and support services for family caregivers of OPwD.

## Data Availability

The interview transcripts are not available due to privacy issues, but are available from the corresponding author on reasonable request.

## Abbreviations

OPwD: Older People with Dementia
